# Machine learning–derived serum carbon dioxide trajectories in predicting prognosis of patients with acute aortic dissection

**DOI:** 10.4103/mgr.MEDGASRES-D-25-00361

**Published:** 2026-08-19

**Authors:** Yong-Bo Zhao, Xin Zhang, Meng-Xin Zhu, Yi-Xuan Wang, Wen-Tao Ding, Xue-Sen Zhao, Dong Ma, Zi-Qiang Tian

**Affiliations:** 1 Department of Thoracic Surgery, The Fourth Hospital of Hebei Medical University, Shijiazhuang, Hebei Province, China; 2 Department of Biochemistry and Molecular Biology, Key Laboratory of Neural and Vascular Biology, Ministry of Education, and Hebei Key Laboratory of Cardiovascular Homeostasis and Aging, Hebei Medical University, Shijiazhuang, Hebei Province, China; 3 School of Public Health, North China University of Science and Technology, Tangshan, Hebei Province, China

**Keywords:** acute aortic dissection, LASSO regression, machine learning, model, postoperative outcome, preoperative prediction, PvCO_2_, SHAP, trajectories analysis, website establishment

## Abstract

Venous partial pressure of carbon dioxide (PvCO_2_) indicates postoperative adverse outcomes in patients with acute aortic dissection, and its trajectories over time affecting postoperative adverse outcomes remain unclear. This study aimed to investigate the relationship between PvCO_2_ trajectories and postoperative adverse outcomes, as well as clinical application through group-based trajectory modeling and machine-learning algorithms. This retrospective cohort study analyzed data from 642 patients with acute aortic dissection treated at the Fourth Hospital of Hebei Medical University from 2019 to 2024, to determine postoperative adverse outcomes, including 6-month mortality and complications. Three PvCO_2_ tests within the first 72 hours of admission were used to analyze PvCO_2_ trajectories through group-based trajectory modeling. To diagnose high-risk PvCO_2_ trajectories in emergency settings, least absolute shrinkage and selection operator regression was conducted to select key features, which were consequently integrated into predictive models using multiple machine-learning methods. Finally, a web-based interpretable website was developed for predicting PvCO_2_ trajectories in patients with acute aortic dissection. Overall, 642 patients were classed into four PvCO_2_ trajectories (T1, persistent decrease from a medium to a low level; T2, stable low level; T3, persistent decrease from a high to a low level; T4, stable midlevel), showing distinct patterns. After adjustment for other covariates, postoperative adverse outcomes among these groups showed significant differences, with a higher proportion of postoperative adverse outcomes in the T3 group. Subsequently, upon Shapley additive explanations analysis, 17 features with importance sequence were identified (hemoglobin, direct bilirubin, monocyte, adenosine deaminase, drinking, anion gap, chloride, platelet, lymphocyte, albumin/globulin ratio, calcium, alanine aminotransferase, α-L-fucosidase, D-dimer, antihypertensive drugs, and smoking), which were integrated into the model for predicting T3. The optimal machine-learning model was established for predicting postoperative adverse outcomes. Finally, a web-based interpretable model was developed to predict patients with acute aortic dissection and high risk of postoperative adverse outcomes. This study uncovers heterogeneity in PvCO_2_ trajectory, highlighting that decreased PvCO_2_ is associated with worse prognosis. A web-based tool for the prediction of acute aortic dissection patients at high risk of postoperative adverse outcomes was constructed to facilitate clinical application.

## Graphical abstract

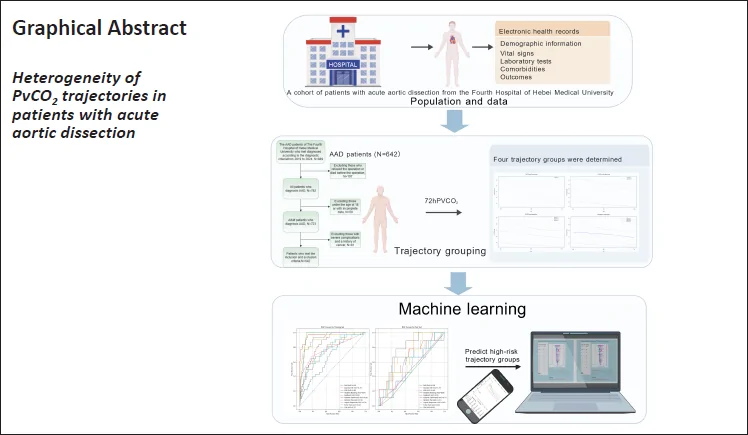

## Introduction

Acute aortic dissection (AAD) is a severe cardiovascular emergency characterized by aortic wall separation, forming a false lumen that can compromise vital organ perfusion. This condition is associated with high mortality rates owing to rapid progression and severe complications, such as aortic rupture and acute kidney injury.^1^,^2^ Despite surgical advancements, postoperative in-hospital mortality remains high, ranging from 7% to 27%.^3^,^4^ Previous studies from our group have identified several variables associated with mortality, such as blood chloride (CL), sodium, glucose, carbon dioxide (CO_2_) binding capacity, and α-hydroxybutyric acid.^5^,^6^

In clinical practice, the venous partial pressure of CO_2_ (PvCO_2_) is a low-cost and time-saving auxiliary diagnostic tool that reflects acid–base homeostasis. A previous study has established an association between PvCO_2_ levels at hospital admission in patients with AAD and outcomes such as all-cause mortality and adverse events.^5^ Similarly, recent reports have indicated that high lactate levels are a risk factor for complications in acute type AAD, such as postoperative acute kidney injury.^7^,^8^,^9^ Notably, Tan et al.^10^ reported that low serum bicarbonate levels upon admission in patients with AAD are associated with higher short- and long-term mortality rates, suggesting that bicarbonate levels can serve as a prognostic indicator. In a 2022 study by our research group, a correlation was found between short-term postoperative mortality and CO_2_ binding capacity (CO_2_CP) in patients with AAD, with lower CO_2_CP levels indicating a higher mortality risk.^11^ Both PvCO_2_ and CO_2_CP are integral components of the body’s acid–base balance, with PvCO_2_ reflecting the partial pressure of dissolved CO_2_ in the blood and CO_2_CP indicating the total amount of CO_2_ in the blood, including both dissolved and bicarbonate forms. These parameters provide complementary information on the patient’s metabolic and respiratory status, making them valuable for assessing the overall acid–base homeostasis in patients with AAD. However, these studies are limited by factors such as varied time points for PvCO_2_ measurement and individual differences in treatment responses, which compromise the accuracy of prognostic assessments.^12^ Therefore, dynamic data collection throughout the disease course is necessary. Fitting novel trajectory models of PvCO_2_ levels may make it possible to explore meaningful heterogeneous PvCO_2_ trajectories in patients with AAD, thereby enhancing the prognostic value of these parameters.

This study aimed to enhance the personalized treatment of patients with AAD by elucidating dynamic changes in PvCO_2_ using group-based trajectory modeling.^13^,^14^ By accurately delineating heterogeneity in PvCO_2_ trajectories, we seek to improve the precision of AAD management and align therapeutic interventions with individual clinical contexts and prognostic profiles. This effort is crucial for optimizing patient outcomes. To achieve this, the optimal machine-learning prediction model was selected based on performance metrics, ensuring robustness and reliability in predicting high-risk PvCO_2_ trajectory groups. Shapley Additive exPlanations (SHAP) were further utilized to interpret model predictions, offering insights into feature importance for adverse outcomes. Additionally, a user-friendly prediction website was developed to provide real-time, actionable applications for clinicians, thereby enhancing the quality of care.

## Methods

### Study population

This retrospective cohort study included patients admitted to the Fourth Hospital of Hebei Medical University between 2019 and 2024. Patients meeting the Chinese expert consensus on AAD diagnosis and treatment were included.^15^ Conversely, individuals aged < 18 years, patients undergoing surgical treatment without serious complications at admission, those with incomplete information, and patients with a history of cancer were excluded. For trajectory model construction, participants with fewer than three PvCO_2_ tests within the first 3 days postadmission were excluded. Ultimately, 642 participants were included in the analysis. This study was conducted in accordance with both the *Declaration of Helsinki* and the International Conference on Harmonization Guidelines for Good Clinical Practice. The study protocols were approved by the Ethics Committee of Fourth Hospital of Hebei Medical University (approval No. 2021k7359) and Hebei Medical University (approval No. P2022041) on March 2, 2022, and obtained all appropriate consent forms from the patients. The reporting of this study adheres to the Strengthening the Reporting of Observational Studies in Epidemiology statement.^19^

### Variable measurements and outcomes

The longitudinal PvCO_2_ trajectory within 3 days after admission was included and summarized each day. If there were multiple measurements in 1 day, the mean value was calculated for analysis.

Patient’s baseline characteristics, including demographic data (age, sex, medical history, smoking, drinking, and length of hospitalization), and initial postadmission measurements of vital signs and laboratory tests were extracted. The primary endpoints of this study were 6-month mortality and all-cause mortality following surgical treatment. Secondary endpoints included the occurrence of postoperative complications, such as heart failure, gastrointestinal bleeding, liver injury, lung injury, kidney injury, shock, multiple organ injury, and the need for invasive ventilator-associated breathing.

### Group-based trajectory model

Group-based trajectory modeling, a semiparametric model for analyzing longitudinal data,16 was employed to examine variations in PvCO_2_ trajectory from 24- to 72-hour postadmission among patients, each characterized by homogeneous trajectory profiles regarding their PvCO_2_ trajectory. The fitting effect of the final model was evaluated using the following indicators: (i) Bayesian information criterion (BIC, a value closer to 0 indicates a better model fit), (ii) number of members per group (each group should contain > 5% of the total participants to ensure relevance and statistical significance), (iii) average posterior probability (AvePP, reflecting the degree of conformity between the subgroup members’ trajectories and the assigned trajectory, with values typically > 0.7 considered acceptable), and (iv) visual distinctiveness of trajectory paths (visually distinct paths confirm practical distinctions among groups). Model fit was rigorously evaluated using the BIC and AvePP. A close-to-zero BIC value and AvePP > 0.7 indicated a robust model fit, suggesting that the identified trajectories are both significant and meaningful in representing the underlying patterns of PvCO_2_ trajectory changes within the population studied.^17^,^18^

### Construction of a classifier for the identification of special acute aortic dissection subclasses

A subgroup of patients with AD exhibiting a high-level decline in PvCO_2_ levels recorded the highest mortality rate. Given the complex fluctuation patterns of 72-hour PvCO_2_ levels in patients with AAD, distinguishing this high-risk subgroup at baseline PvCO_2_ levels posed a challenge. To this end, research participants were selected using a simple random sampling method, and they were divided into a training set (*n* = 449) and a test set (*n* = 193) in a 7:3 ratio. Then, the least absolute shrinkage and selection operator (LASSO) regression analysis was conducted to identify the key features that correlated with the PvCO_2_ trajectory in the training set. These features were integrated into predictive models trained by multiple algorithms, such as the support vector machine, Gaussian Naive Bayes, K-nearest neighbors, gradient boosting, AdaBoost, XGBoost, decision tree, logistic regression, extra trees, and linear discriminant analysis, and hyperparameter optimization was performed via grid search combined with 10-fold cross-validation. Model performance was evaluated by calculating the area under the receiver operating characteristic curve (AUC) between the two sets. Additionally, SHAP was employed for model interpretation. Finally, to enhance the clinical utility, the xgb.plot.importance function in RStudio was used to rank the importance of variables through numerical analysis. A web-based tool (https://t3trajectoryprediction.shinyapps.io/documention/) was developed using the R Shiny package. The tool allows users to input key clinical variables and obtains real-time individualized risk predictions for belonging to the T3 trajectory. The Shiny application was built with a standard UI-server structure: the UI collects user inputs via interactive controls, and the server processes these inputs using the pre-trained XGBoost model to generate predicted probabilities.

### Statistical analysis

R 4.5.0 (https://www.r-project.org) and Python 3.6 (https://www.python.org) were used for statistical analysis. In assessing the significance of between-group differences for continuous variables, Student’s *t*-test was utilized for normally distributed data and the Wilcoxon–Mann–Whitney rank sum test for non-normally distributed data. Categorical variables were summarized as frequencies and percentages, with the chi-square test applied to evaluate significance among the different PvCO_2_ trajectory groups. Continuous variables with non-normal distribution were compared among multiple groups using the Kruskal–Wallis *H* test and presented as median (interquartile range). Categorical variables were compared using the chi-square test and presented as frequencies (percentages). All statistical tests were two-tailed, and *P* values < 0.05 were considered statistically significant. To address the issues of missing data within our covariates, latent class trajectory-based multiple imputation was employed, thereby ensuring the integrity of the subsequent analyses.

## Results

### Baseline characteristics of patients with acute aortic dissection

Initially, 889 patients were diagnosed with AD, and ultimately, 642 patients met the inclusion and exclusion criteria (**Figure 1**). Based on the BIC and AvePP results (**Table 1**), PvCO_2_ trajectories were divided into four segments: T1 (persistent decrease from a medium to a low level), where PvCO_2_ rapidly decreases from a medium to a low level over time; T2 (stable low level), where PvCO_2_ remains at a low level; T3 (persistent decrease from a high to a low level), where PvCO_2_ rapidly decreases from a high to a low level over time; T4 (stable medium level), where PvCO_2_ remains at a moderate level (**Figure 2**). Subsequently, the baseline characteristics of patients with AAD from different partial pressure of arterial carbon dioxide trajectory groups on admission are presented in **Table 2**. Some variables compared in four groups demonstrated significant differences, such as Stanford A/B and surgical history.

**Figure 1:**
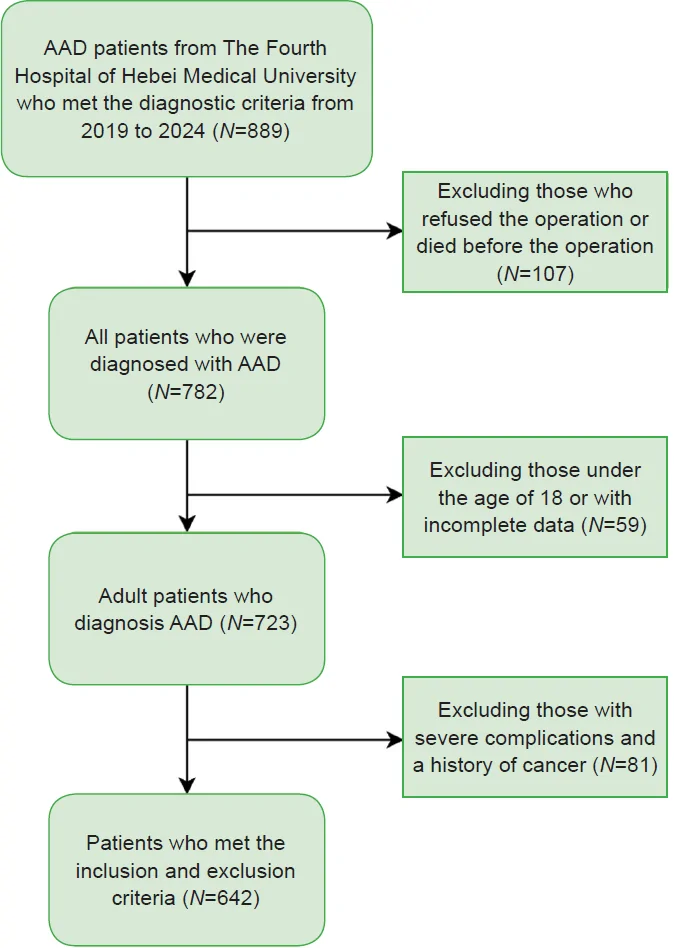
Flowchart of patient selection process. A total of 889 AAD patients were screened. After excluding 107 patients who refused surgery/died preoperatively, 59 patients aged < 18 years or with incomplete data, and 81 patients with severe complications/cancer history, 642 patients were included in the final analysis. AAD: Acute aortic dissection.

**Figure 2:**
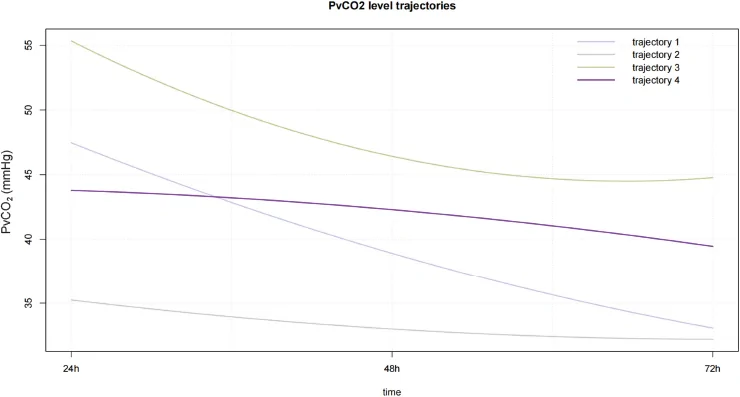
Group-based trajectory modeling of serum carbon dioxide levels during the first 72 hours after admission to the hospital. Trajectory 1 (T1): consistently low PvCO_2_ levels; Trajectory 2 (T2): moderate levels with a slight increase; Trajectory 3 (T3): elevated levels with a gradual decrease; Trajectory 4 (T4): persistently high PvCO_2_ levels. These trajectories reflect distinct patterns of change in serum carbon dioxide during the early phase of hospitalization. AAD: Acute aortic dissection; PvCO_2_: venous partial pressure of carbon dioxide.

**Table 1:** Model fit indices and average posterior probabilities for latent class trajectory analysis

	aic	bic	caic	ssbic	hqic	T1_avepp	T2_avepp	T3_avepp	T4_avepp
T1	13126.40	13143.09	13146.09	13133.56	13132.54	1	0.9421790	0.9131611	0.7832773
T2	12810.28	12854.79	12862.79	12829.37	12826.66		0.8772984	0.9044792	0.9220148
T3	1252727	12599.59	12612.59	12558.29	12553.88			0.8720719	0.8738560
T4	12473.64	12579.34	12598.34	12518.98	12512.52				0.8874874

Trajectory 1 (T1): consistently low PvCO_2_ levels; Trajectory 2 (T2): moderate levels with a slight increase; Trajectory 3 (T3): elevated levels with a gradual decrease; Trajectory 4 (T4): persistently high PvCO_2_ levels. These trajectories reflect distinct patterns of change in serum carbon dioxide during the early phase of hospitalization. Lower values of AIC, BIC, caic, ssBIC, and HQIC indicate better model fit. Average posterior probabilities > 0.7 suggest good classification accuracy for the corresponding trajectory group. AIC: Akaike information criterion; BIC: Bayesian information criterion; caic: consistent Akaike information criterion; ssBIC: sample-size adjusted Bayesian information criterion; HQIC: Hannan-Quinn information criterion; T1–T4_avepp: average posterior probability for trajectorys 1–4.

**Table 2:** Baseline characteristics of the sepsis patients with different PaCO_2_ trajectories at admission

	Overall (*n* = 642)	T1 (*n* = 42)	T2 (*n* = 253)	T3 (*n* = 40)	T4 (*n* = 307)	*P*
Sex						0.306
Female	482(75.1)	36(85.7)	185(73.1)	32(80.0)	229(74.6)	
Male	160(24.9)	6(14.3)	68(26.9)	8(20.0)	78(25.4)	
Stanford						< 0.001
A	506(78.8)	19(45.2)	175(69.2)	36(90.0)	276(89.9)	
B	136(21.2)	23(54.8)	78(30.8)	4(10.0)	31(10.1)	
Surgical history (%)	136(21.2)	2(4.8)	59(23.3)	4(10.0)	71(23.1)	0.011
Hypertension	456 (71.0)	29 (69.0)	175 (69.2)	31 (77.5)	221 (72.0)	0.692
Hypertension grading						< 0.001
0	241(37.5)	33(78.6)	93(36.8)	12(30.0)	103(33.6)	
1	42(6.5)	1(2.5)	14(5.0)	4(10.0)	23(7.5)	
2	96(15.0)	3(7.1)	39(15.4)	5(12.5)	49(16.0)	
3	263(41.0)	6(14.3)	106(041.9)	19(47.5)	132(43.0)	
Past history (%)						
AHD	303 (47.2)	7 (16.7)	131 (51.8)	24 (60.0)	141 (45.9)	< 0.001
DM	24(3.7)	3(7.1)	10(4.0)	1(2.5)	10(3.3)	0.359
CHD	32 (5.0)	1(2.4)	17(6.7)	3(7.5)	11(3.6)	0.268
Smoking	300(46.7)	23(54.8)	113(44.7)	24(60.0)	140(45.6)	0.212
Drinking	319(49.7)	26(61.9)	116(45.8)	28(70.0)	149(48.5)	0.013
BMI (kg/m^2^)	26.17(24, 29.41)	27(24.75, 29.25)	26(24, 29.07)	27.91(24.34, 29.75)	26.12(23.55, 30)	0.686
Duration of hospitalization	18(9, 27)	20(14,31.5)	16(8, 25)	16(6, 23.75)	21(12, 30)	< 0.001
Age (yr)	53(45, 61.25)	52(46.75, 62.25)	54(45, 62)	51.5(42, 58.75)	53(45, 61)	0.417
SBP (mmHg)	143(127, 162)	138(120, 157.5)	143(129, 163)	145.5(122.75,166.25)	143(127, 161)	0.445
DBP (mmHg)	80(68, 92)	77(68.75, 90.5)	80(71, 94)	74.5(66, 89.5)	79.24(67, 92)	0.402
Na (mM)	139(137, 140)	138(136, 140)	138(137, 140)	138.5(136, 141)	139(137, 141)	0.447
K (mM)	3.8(3.5, 4.1)	4(3.65, 4)	3.8(3.5, 4.2)	3.9(3.35, 4.1)	3.8(3.5, 4.04)	0.126
CI (mM)	105(103, 107)	106(103, 108)	105(102.1, 107)	104(101.25, 106)	105.00(103, 107)	0.044
PaCO_2_ (mmHg)	24(22, 25.8)	22.85(20, 24.15)	24(22.3, 25.7)	23.7(21.23, 25.75)	24(22, 26)	0.014
Ca (mg/dL)	2.22(2.14, 2.29)	2.17(2.1, 2.25)	2.23(2.15, 2.3)	2.18(2.11, 2.28)	2.22(2.15, 2.3)	0.017
AG (mM)	13.2(11.9, 15.4)	13.8(12.4, 15.76)	13.2(11.8, 15.2)	14.25(12.73, 17.43)	13(11.8, 15.4)	0.025
P (mg/dL)	1.14(0.97, 1.30)	1.12(0.91, 1.21)	1.15(0.97, 1.32)	1.15(1.03, 1.33)	1.11(0.98, 1.27)	0.479
Mg (mg/dL)	0.84(0.77, 0.89)	0.87(0.78, 0.91)	0.83(0.77, 0.89)	0.82(0.77, 0.88)	0.83(0.76, 0.89)	0.425
ALT (U/L)	23.55(16.8, 35.65)	22.05(17.45, 31)	23.2(16.3, 40.25)	25.8(18.4, 48.77)	23.5(16.9, 34.9)	0.322
AST (U/L)	24.75(19, 40.13)	23.15(18.45, 49.25)	25.7(19.5, 43.95)	30.8(23.65, 44.45)	23.5(18.4, 35.6)	0.012
PA (mg/dL)	226.6(181.5, 278.7)	203.95(165.7,277.93)	225.4(181.5, 276.1)	210.4(168.63,295.83)	232.1(187.7, 285.6)	0.563
TP (g/dL)	62.35(57.3, 66.1)	59(52.48, 64.8)	61.6(56.5, 65.5)	62.65(57.68, 66.5)	63.1(58.6, 66.4)	0.009
ALB (g/dL)	39.3(35.78, 41.7)	38(33.1, 40.35)	39.2(35.4, 41.65)	38.6(34.9, 41.73)	39.5(37.1, 41.9)	0.069
GLB (g/dL)	23.3(20.35, 26.1)	22.7(18.83, 25.1)	23.5(20.35, 26.9)	24(20.35, 26.75)	23.25(20.6, 25.93)	0.174
A/G	1.68(1.46, 1.92)	1.71(1.49, 1.99)	1.65(1.44, 1.88)	1.68(1.39, 1.91)	1.69(1.48, 1.91)	0.650
TBIL (mg/dL)	14.75(11.2, 21.35)	19.95(13.08, 25.6)	14(10.75, 19.35)	17.5(12.65, 23.33)	14.7(11.3, 21.5)	0.004
DBIL (mg/dL)	5.3(4, 6.92)	5.15(3.68, 8.47)	5.1(4, 6.9)	6.3(4.85, 8.18)	5.3(3.9, 6.9)	0.194
IBIL (mg/dL)	9.9(6.2, 14.02)	12.4(7.25, 18.53)	8.7(6, 12.65)	10.15(6.2, 14.6)	10.2(6.5, 14.5)	< 0.001
ALB (g/dL)	71.05(58, 93.58)	65.4(50.1, 83.93)	72.2(58.12, 94.95)	67(55.05, 91.05)	71.2(58.3, 93.5)	0.159
GGT (U/L)	29.1(18.75, 50.1)	27.15(19.6, 51.33)	28.2(18.85, 51.55)	32(21.7, 45.28)	30.15(18.18, 50.45)	0.794
ADA (U/L)	9.2(7.2, 11.9)	8.85(6.88, 12.8)	8.9(7.35, 11.75)	10.45(7.78, 14.15)	9.3(7.2, 11.9)	0.191
AFU (U/L)	21.9(17, 26)	20.15(16.83, 24)	22(17.05, 25.8)	22.4(17.48, 27.68)	22(16.9, 26)	0.417
CHE (U/L)	7644(6109.8, 9067.35)	7445.15(5514.95, 9046.05)	7465(6090.5, 8962.1)	7266(5919.75,8639.93)	7877.6(6370, 9242.2)	0.162
CREA (mg/dL)	76.1(61, 98.3)	78.2(64.02, 111.82)	74.2(58.65, 97.75)	81.7(66.88, 112.2)	76.1(61.1, 95.9)	0.233
Urea (mg/dL)	6.2(5, 8)	6.4(5.1, 8.95)	6(4.8, 7.86)	6.15(5.2, 8.1)	6.2(5.1, 7.82)	0.394
GLU (mg/dL)	6.99(5.95, 8.29)	6.71(5.83, 9.37)	6.89(5.87, 8.08)	7.13(6.31, 8.59)	7.04(6.06, 8.3)	0.406
UA (mg/dL)	359.25(278.82, 439)	347.5(256.2, 455.5)	352.8(279.55,418.2)	421.4(299.13,490.15)	359.4(278, 444)	0.076
Tn (ng/L)	0.012(0.012, 0.081)	0.012(0.011, 0.65)	0.012(0.012, 0.81)	0.019(0.012, 0.11)	0.012(0.012, 0.086)	0.663
WBC (×10^9^/L)	11.75(9, 14.23)	11.88(7.78, 16.13)	10.72(8.47, 13.87)	13.21(9.73, 16.55)	12.28(9.33, 14.3)	0.001
NEU (×10^9^/L)	9.97(7.4, 12.56)	9.74(6.13, 14.29)	9.4(6.62, 11.87)	11.15(8.42, 12.7)	10.62(7.6, 12.71)	0.004
LYM (×10^9^/L)	0.57(0.41, 1.23)	0.89(0.68, 1.27)	0.90(0.63, 1.25)	0.83(0.56, 1.45)	0.79(0.57, 1.18)	0.096
MONO (×10^9^/L)	0.57(0.41, 0.81)	0.61(0.42, 0.94)	0.54(0.41, 0.78)	0.68(0.44, 1.02)	0.58(0.39, 0.8)	0.129
EOS (×10^9^/L)	0.01(0, 0.03)	0.01(0, 0.035)	0.01(0, 0.04)	0(0, 0.03)	0.01(0, 0.02)	0.094
BASO (×10^9^/L)	0.01(0.01, 0.02)	0.015(0.008, 0.02)	0.01(0.01, 0.02)	0.01(0, 0.03)	0.01(0, 0.02)	0.496
RBC (×10^12^/L)	4.23(3.81, 4.64)	4.2(3.8, 4.65)	4.2(3.78, 4.64)	4.21(3.91, 4.48)	4.26(3.83, 4.65)	0.726
HGB (g/dL)	143(131.6, 143)	126.8(115.72,147.08)	131(116, 143)	134(125.5, 138)	132.9(117, 143)	0.749
HCT (%)	0.43(0.39, 0.43)	0.39(0.34, 0.44)	0.39(0.35, 0.42)	0.39(0.37, 0.42)	0.40(0.35, 0.43)	0.432
PLT (×10^9^/L)	169(132, 210)	181(131.75, 215)	171(135, 214)	157(128, 200.75)	168(131, 204)	0.481
PT (s)	12.4(11.6, 13.4)	12.5(11.6, 13.58)	12.4(11.5, 13.4)	12.4(11.7, 13)	12.3(11.6, 13.4)	0.823
FIB (g/L)	2.55(1.97, 3.32)	2.45(1.88, 3.33)	2.59(2.15, 3.38)	2.55(1.94, 3.31)	2.46(1.87, 3.29)	0.189
DD (mg/L)	2.29(0.92, 7.56)	2.27(0.87, 9.1)	2.06(0.97, 6.83)	2.81(1.15, 9.48)	2.31(1.01, 8.36)	0.381

For continuous variables with non-normal distribution, the Kruskal–Wallis *H* test was used for comparisons among multiple groups. For categorical variables, the chi-square test was applied. A/G: Albumin to globulin ratio; ADA: adenosine deaminase; AFU: alpha fucosidase; AG: anion gap; ALB: albumin; ALP: alkaline phosphatase; ALT: alanine aminotransferase; AST: aspartate aminotransferase; BMI: body mass index; Ca: calcium; CHE: cholinesterase; Cl: chlorine; CO_2_: carbon dioxide; CREA: creatinine; DBIL: direct bilirubin; DBP: diastolic blood pressure; DD: D-dimer; EOS: eosinophil count; FIB: fibrinogen; GGT: gamma-glutamyltranspeptidase; GLB: globulin; GLU: glucose; HCT: hematocrit; HGB: hemoglobin; IBIL: indirect bilirubin; K: potassium; LYM: lymphocyte count; Mg: magnesium; MONO: monocyte count; Na: sodium; NEU: neutrophil count; P: phosphorus; PA: prealbumin; PaCO_2_: partial pressure of arterial carbon dioxide; PLT: platelet count; PT: prothrombin time; RBC: red blood cell count; SBP: systolic blood pressure; TBIL: total bilirubin; Tn: troponin; TP: total protein; UA: uric acid; UREA: urea; WBC: white blood cell count.

### Relationship between postoperative adverse outcomes and venous partial pressure of carbon dioxide trajectories

The highest mortality rate was recorded in the T3 group, characterized by the high incidence of heart failure, gastrointestinal bleeding, and liver injury, as well as an increased requirement for mechanical ventilation (**Table 3**). Moreover, Kaplan–Meier survival analysis revealed the highest mortality rate in the T3 group, intermediate rates in the T2 and T4 groups, and the lowest rate in the T1 group, each of which exhibited a marked decrease in mortality risk (all *P* < 0.05; **Figure 3**).

**Figure 3:**
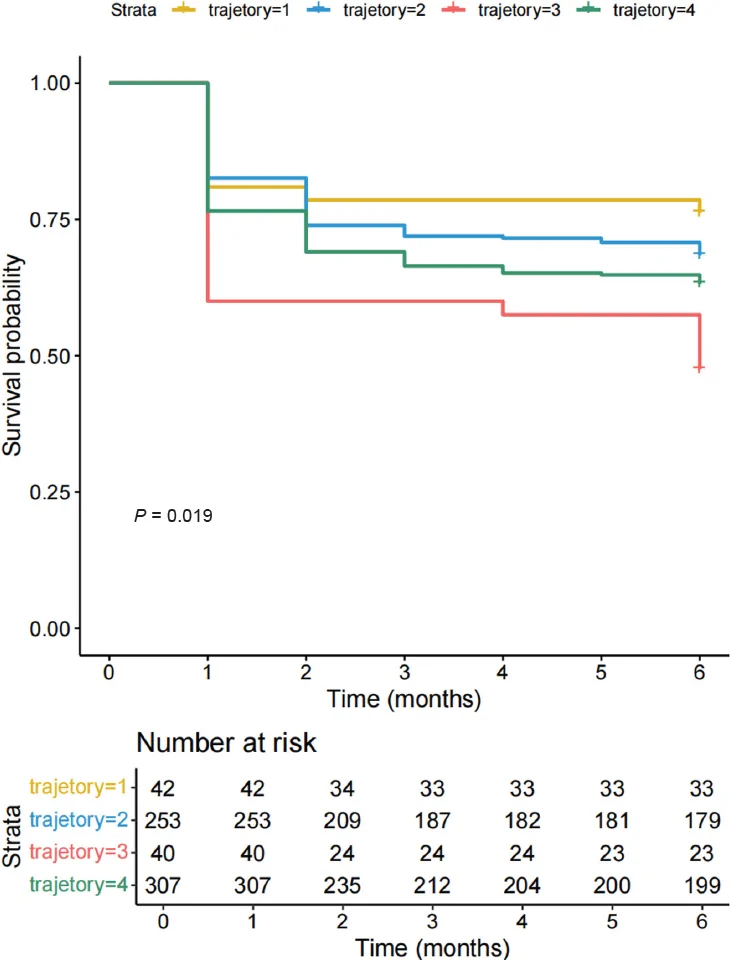
Association between serum carbon dioxide trajectories and 6-month mortality. Patients with persistently high PvCO_2_ (T4) had the lowest survival, while those with consistently low PvCO_2_ (T1) had the highest survival (log_2_rank *P* < 0.05). Trajectory 1 (T1): consistently low PvCO_2_ levels; Trajectory 2 (T2): moderate levels with a slight increase; Trajectory 3 (T3): elevated levels with a gradual decrease; Trajectory 4 (T4): persistently high PvCO_2_ levels. These trajectories reflect distinct patterns of change in serum carbon dioxide during the early phase of hospitalization. AAD: Acute aortic dissection; PvCO_2_: Venous partial pressure of carbon dioxide.

**Table 3:** Clinical outcomes of the patients with different PvCO_2_ trajectories

	Overall (*n* = 642)	T1 (*n* = 42)	T2 (*n* = 253)	T3 (*n* = 40)	T4 (*n* = 307)	*P*
Death	224 (34.9)	10 (23.8)	80 (31.6)	21 (52.5)	113 (36.8)	0.024
Heart failure	82(12.8)	4(9.5)	9(7.5)	9(22.5)	50(16.3)	0.004
Gastrointestinal bleeding	32(5.0)	2(4.8)	13(5.1)	6(15.0)	11(5.0)	0.021
Liver injury	33(5.1)	1(2.3)	7(2.8)	5(12.5)	20(6.5)	0.014
Ventilator-assisted breathing						<0.001
Non-invasive breathing	387(60.3)	38(90.5)	126 49.8)	22(55.0)	201(65.5)	
Invasive breathing	255(39.7)	4(9.5)	127(50.2)	18(45.0)	106(34.5)	
Shock	36(5.6)	1(2.4)	17(6.7)	2(5.0)	16(5.2)	0.674
Lung injury	64(10.0)	3(7.1)	24(9.5)	2 (5.0)	35(11.4)	0.323
MODS	54(8.4)	3(7.1)	26(10.3)	6(15)	19(6.2)	0.162
AKI	90(14)	3(5.9)	32(12.6)	8(20.0)	47(15.3)	0.301

Data are expressed as number (percentage). For continuous variables with non-normal distribution, the Kruskal–Wallis *H* test was used for comparisons among multiple groups. For categorical variables, the chi-square test was applied. AKI: Acute kidney injury; MODS: multiple organ dysfunction syndrome; PvCO_2_: venous partial pressure of carbon dioxide.

With T3 as the reference, logistic regression analysis showed that the odds ratios (ORs) of T1, T2, and T4 were 0.28 (95% confidence interval (CI) 0.11–0.71), 0.42 (95% CI 0.21–0.82), and 0.53 (95% CI 0.27–1.02), respectively, in model 1 without adjustment for covariates (**Table 4**). After adjustment for sex, age, systolic blood pressure, diastolic blood pressure, and body mass index in model 2, the ORs of T1, T2, and T4 were 0.27 (95% CI 0.10–0.70), 0.41 (95% CI 0.21–0.82), and 0.51 (95% CI 0.26–1.00), respectively. Despite further adjustment for sex, age, systolic blood pressure, diastolic blood pressure, body mass index, smoking history, drinking history, hypertension, diabetes, and coronary heart disease in model 3, the mortality risk of T3 remained significant compared with that of T1 (OR 0.28, 95% CI 0.10–0.72), T2 (OR 0.39, 95% CI 0.19–0.78), and T4 (OR 0.50, 95% CI 0.25–0.98). These results suggested that the T3 group exhibited the highest mortality risk among all subgroups.

**Table 4:** Logistic regression models for postoperative adverse outcomes and PvCO_2_ trajectories

PvCO_2_ trajectories	Model 1		Model 2		Model 3	
		
	OR(95%CI)	*P*	OR(95%CI)	*P*	OR(95%CI)	*P*
T3	1	Reference	1	Reference	1	Reference
T1	0.28(0.11–0.71)	<0.001	0.27(0.10–0.70)	0.008	0.28(0.10–0.72)	0.009
T2	0.42(0.21–0.82)	0.011	0.41(0.21–0.82)	0.011	0.39(0.19–0.78)	0.008
T4	0.53(0.27–1.02)	0.058	0.51(0.26–1.00)	0.050	0.50(0.25–0.98)	0.043

T1: Persistent decrease from medium to low level; T2: stable low-level; T3: persistent decrease from high to low level; T4: stable mid-level. Model 1: Unadjusted rough model; Model 2: adjusted model (adjusted for variables from sex, age, systolic blood pressure, diastolic blood pressure, BMI); Model 3: adjusted model (adjusted for variables from sex, age, systolic blood pressure, diastolic blood pressure, BMI, smoking history, drinking history, hypertension, diabetes, coronary heart disease). AAD: Acute aortic dissection; BMI: body mass index; CI: confidence interval; OR: odds ratio; PvCO_2_: venous partial pressure of carbon dioxide.

### LASSO regression important feature variable screening and establishment of multiple model comparisons

LASSO regression analysis results showed that 17 characteristic variables are associated with T3 mortality: hemoglobin, direct bilirubin (DBIL), monocyte, adenosine deaminase, drinking, anion gap, CL, platelet, lymphocyte, albumin/globulin ratio, calcium, alanine aminotransferase, α-L-fucosidase, D-dimer, antihypertensive drugs, and smoking, with 10 times cross-validation and optimal value of lambda.1se = 0.01662473 (**Figure 4**). Then, 10 ML algorithms were applied to predict the mortality risk in patients with AAD, accompanied by comparisons of predictive performance among them. As shown in **Figure 5**, the XGBoost model exhibited the optimum performance, with AUCs of 0.83 (95% CI 0.81–0.85) in the training set and 0.77 (95% CI 0.74–0.82) in the test set.

**Figure 4:**
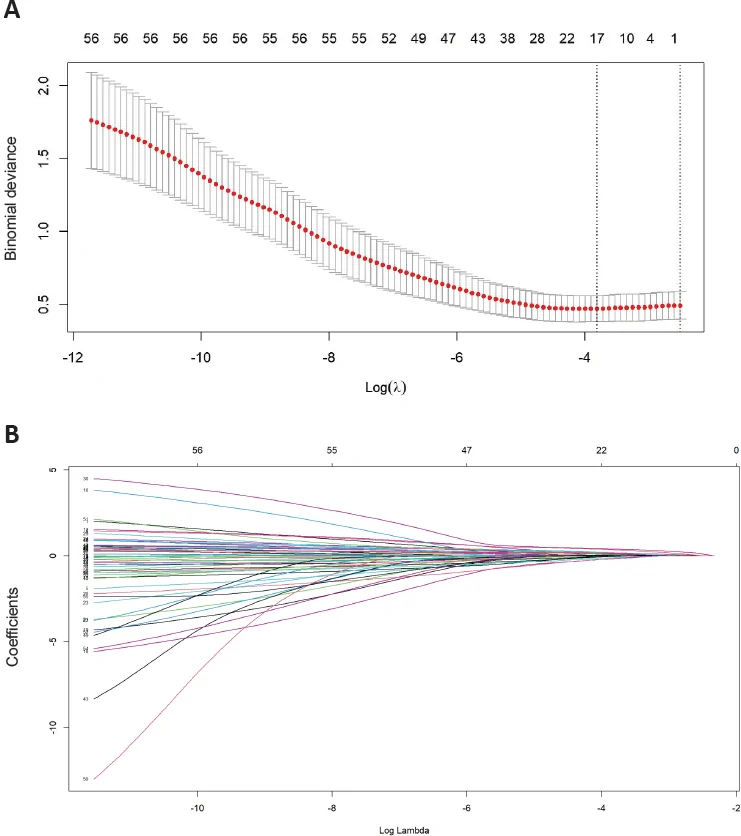
Selection of characteristic factors for the T3 trajectory group using LASSO regression. (A) LASSO coefficient path profiles. Each colored line corresponds to a specific clinical variable. The numbers listed at the top indicate the number of non-zero coefficients (variables) retained at the corresponding log(λ) value. As the penalty increases (moving left), coefficients of less important variables shrink to zero, performing variable selection. (B) Binomial deviance plot for cross-validation. The red points indicate the mean cross-validated error for each λ, with error bars representing the standard deviation. The two vertical dotted lines indicate the optimal λ values selected: the left line (λ.min) corresponds to the minimum deviance, and the right line (λ.1se) corresponds to the most regularized model within 1 standard error of the minimum. The variables selected at λ.1se were included in the final multivariate model. LASSO: Least absolute shrinkage and selection operator.

**Figure 5:**
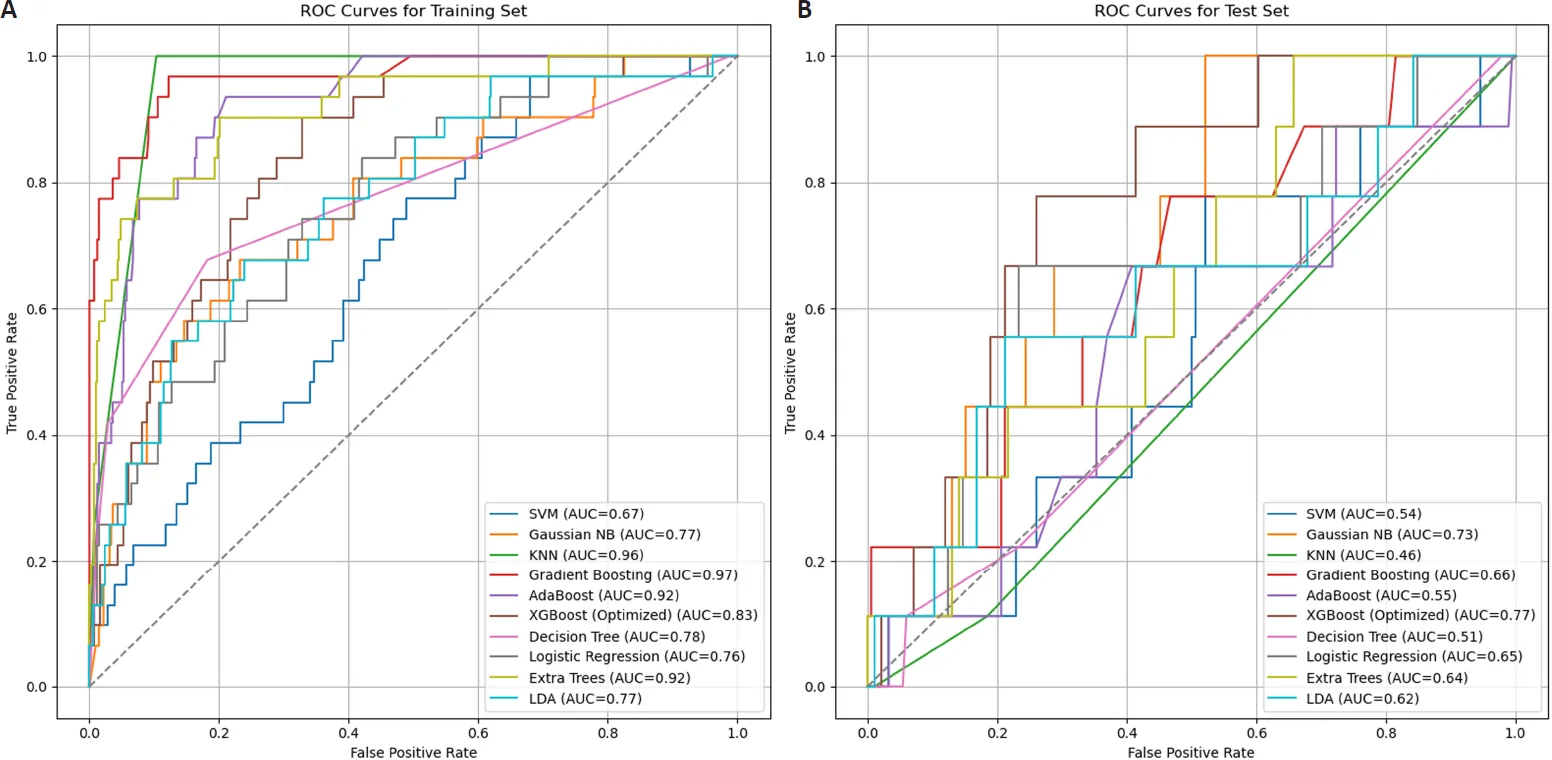
ROC curves of multiple machine learning models for predicting the occurrence of T3 trajectory groups. Ten different algorithms, including SVM, Gaussian NB, KNN, gradient boosting, AdaBoost, optimized XGBoost, decision tree, logistic regression, extra trees, and LDA, were compared. (A) The ROC curves for the training set demonstrate the fitting performance of each model. (B) The ROC curves for the test set evaluate the generalization ability of the models. The higher AUC value indicates better predictive accuracy. AUC: Area under the curve; Gaussian NB: Gaussian Naive Bayes; KNN: K-nearest neighbors; LDA: linear discriminant analysis; ROC: receiver operating characteristic; SVM: support vector machine.

### Model interpretation and prediction website construction

The above results indicate that hemoglobin, DBIL, monocyte, adenosine deaminase, drinking, anion gap, CL, platelet, lymphocyte, albumin/globulin ratio, calcium, alanine aminotransferase, α-L-fucosidase, D-dimer, antihypertensive drugs, and smoking are key features in the XGBoost model in identifying T3 patients. Further, the SHAP summary plot comprehensively displays the contribution of 17 features to the prediction ability (**Figure 6**).

**Figure 6:**
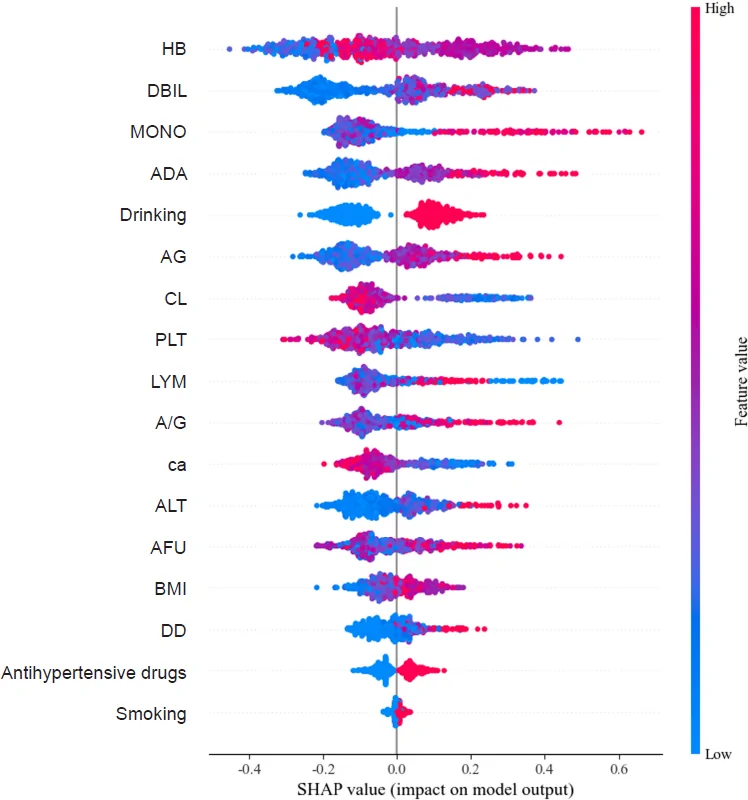
SHAP summary plot showing feature importance for predicting the T3 trajectory group. The positive SHAP values (right side) indicate a higher probability of the patient belonging to the T3 trajectory group, while negative SHAP values (left side) indicate a lower probability. The color gradient represents the value of each feature, with red indicating high values and blue indicating low values. Key predictors identified include HB, DBIL, and MONO counts. A/G: Albumin to globulin ratio; ADA: adenosine deaminase; AFU: alpha fucosidase; AG: anion gap; ALT: alanine aminotransferase; BMI: body mass index; Ca: calcium; Cl: chlorine; DBIL: direct bilirubin; DD: D-dimer; HB: hemoglobin; LYM: lymphocyte count; MONO: monocyte; PLT: platelet count; SHAP: SHapley Additive exPlanations; T3: elevated levels with a gradual decrease.

Finally, a base-web prediction model was constructed for the T3 risk of patients with AAD according to the optimal cutoff value of 0.210 of the prediction model with SHAP explanation. For example, to introduce two independent sample instructions using this website (https://t3trajectoryprediction.shinyapps.io/documention/), the results presented that the T3 risks of patients 1 and 2 were 0.384 and 0.114, respectively (**Figure 7**). The follow-up results ultimately confirmed that patient 1 presented with T3 but not patient 2.

**Figure 7:**
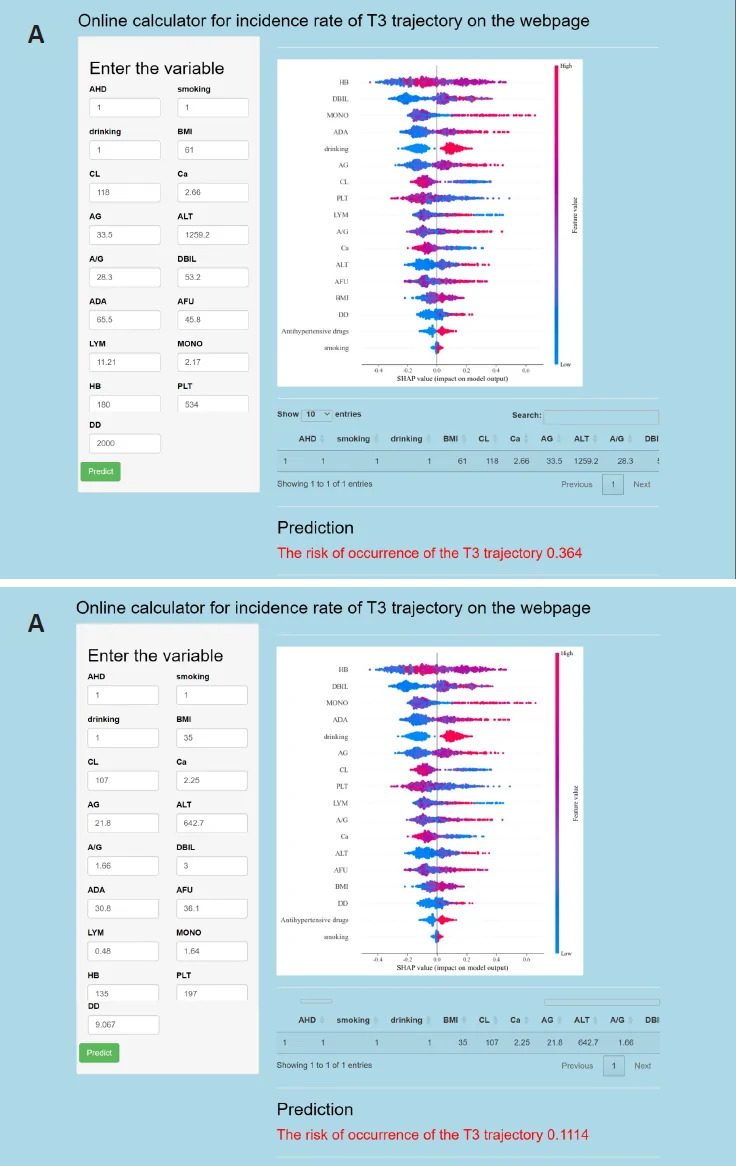
Evaluating AAD surgery death risk with R Shiny package. This web-based tool allows users to input key clinical variables to obtain an individualized risk prediction. (A) Interface display and prediction result for a representative patient who developed the T3 trajectory (high-risk group). (B) Interface display and prediction result for a representative patient who did not develop the T3 trajectory (low-risk group). A/G: Albumin to globulin ratio; AAD: acute aortic dissection; ADA: adenosine deaminase; AFU: alpha fucosidase; AG: anion gap; ALT: alanine aminotransferase; BMI: body mass index; Ca: calcium; Cl: chlorine; DBIL: direct bilirubin; DD: D-dimer; HB: hemoglobin; LYM: lymphocyte count; MONO: monocyte; PLT: platelet count; T3: elevated levels with a gradual decrease.

### Subgroup analysis of T3 by Stanford classification

To address the potential heterogeneity between different anatomical types of AAD, a subgroup analysis stratified by Stanford classification (type A, *n* = 506; type B, *n* = 136) was performed to identify distinct predictors of T3 (representing specific PvCO_2_ evolution patterns). The results revealed significant differences in risk profiles between the two groups (**Additional Table 1** and **Additional Figure 1**). In the Stanford A subgroup, drinking was identified as a significant independent risk factor for T3 (adjusted OR 3.20, 95% CI 1.47–6.96, *P* = 0.003). Additionally, CL levels showed a borderline protective association with T3 (adjusted OR 0.91, 95% CI 0.83–1.00, *P* = 0.048). In contrast, the predictor profile for Stanford B differed substantially. Drinking was not significantly associated with T3 in this subgroup (*P* = 0.723). Instead, DBIL emerged as a significant risk factor (adjusted OR 1.32, 95% CI 1.07–1.63, *P* = 0.011). Consistent with Stanford A, CL maintained a significant protective effect in Stanford B (adjusted OR 0.78, 95% CI 0.62–0.97, *P* = 0.028). Hemoglobin did not demonstrate a significant association with T3 in either subgroup.

**Additional Table 1:** Subgroup analysis of prognostic factors stratified by Stanford A and Stanford B

Variable	Stanford A OR (95% CI)	*P*	Stanford B OR (95% CI)	*P*
**HB**	0.99 (0.98 - 1.02)	0.805	1.06 (0.97 - 1.16)	0.222
**DBIL**	1.02 (0.98 - 1.07)	0.268	**1.32 (1.07 - 1.63)**	**0.011**
**Drinking**	**3.20 (1.47 - 6.96)**	**0.003**	0.66 (0.07 - 6.44)	0.723
**Cl**	**0.91 (0.83 - 1.00)**	**0.048**	**0.78 (0.62 - 0.97)**	**0.028**

95% CI: 95% confidence interval; Cl: chlorine; DBIL: direct bilirubin; HB: hemoglobin; OR: odds ratio.

### Temporal stability of trajectory groups

To further investigate the stability of the PvCO_2_ trajectory model across different admission periods, the cohort was stratified into two distinct time frames: early period (2019–2021, *n* = 257) and later period (2022–2024, *n* = 374). The stratified analysis results (**Additional Table 2**) demonstrated that the relationship between trajectory groups and prognosis remained consistent throughout the study period. All non-reference trajectory groups (T1, T2, and T4) maintained a consistent protective trend (*OR* < 1) compared with the reference group (T3) in periods 1 and 2. Specifically, for T2, the OR was 0.68 (95% CI 0.20–2.32, *P* = 0.53) in period 1 and improved to 0.35 (95% CI 0.16–79, *P* = 0.01) in period 2. T1 showed ORs of 0.33 (*P* = 0.12) in period 1 and 0.29 (*P* = 0.17) in period 2, whereas T4 showed ORs of 0.51 (*P* = 0.26) and 0.58 (*P* = 0.19), respectively. This consistency in the direction of association across the two periods reinforces the robustness of the trajectory model and minimizes the potential bias related to the study duration.

**Additional Table 2:** Sensitivity analysis of PvCO2 trajectories stratified by admission period

PvCO2 trajectories	Period 1 (2019-2021) OR (95% CI)	*P*	Period 2 (2022-2024) OR (95% CI)	*P*
**Reference (T3)**	1.00 (Ref)	Reference	1.00 (Ref)	Reference
**T1**	0.33 (0.08 - 1.33)	0.12	0.29 (0.05 - 1.69)	0.17
**T2**	0.68 (0.20 - 2.31)	0.53	**0.35 (0.16 - 0.79)**	**0.01**
**T4**	0.51 (0.16 - 1.67)	0.26	0.58 (0.26 - 1.31)	0.19

95% CI: 95% confidence interval; OR: odds ratio; PvCO_2_: venous partial pressure of carbon dioxide.

## Discussion

This study employed group-based trajectory modeling to investigate the diverse evolving trajectories of PvCO_2_ levels in patients with AAD. Notably, this research pioneers the examination of the association between dynamic changes in PvCO_2_ levels among adult patients with AAD and their adverse clinical outcomes. Our findings reveal several key insights: initially, four unique PvCO_2_ trajectory groups were identified (T1, persistent decrease from a medium to a low level; T2, stable low level; T3, persistent decrease from a high to a low level; T4, stable midlevel) and their correlation with poor prognosis was assessed. Trend changes in PvCO_2_ levels in 72 hours preceding admission were highly predictive of AAD. The four distinct PvCO_2_ trajectories identified in this study highlight the heterogeneity among patients with AAD, suggesting that differing prognoses are associated with varying altered PvCO_2_ trends. Among these patients with PvCO_2_ trajectories, T1 (PvCO_2_ rapidly decreasing from a medium to a low level over time) exhibited PvCO_2_ fluctuations between 36.2 and 42.2 mmHg, T2 (remaining at a low level) fluctuated between 18.5 and 48.6 mmHg, T3 (decreasing from a high to a low level over time) fluctuated between 30.6 and 74.6 mmHg, and T4 (remaining at a midlevel) varied between 30.8 and 54.6 mmHg. Consistent with a previous study,^11^ the prognosis for these patients worsened as PvCO_2_ levels decreased, and the cumulative postoperative 30-day survival in patients with AAD and low CO_2_CP was the worst. The incidence of AAD, mortality rates, and length of hospital stay increased. The incidence of adverse outcomes progressively increased with decreasing PvCO_2_ levels across the two groups with stable states (T2 and T4).

PvCO_2_, a routine laboratory test, is more accessible in patients, and existing evidence shows that PvCO_2_ is the most accurate single predictor of in-hospital death compared with laboratory tests.^13^ Decreased PvCO_2_ at admission and within the first 24 hours of hospitalization significantly increased the mortality risk in patients with AAD.^5^,^19^ Time-specific or model-dependent receiver operating characteristic also demonstrated the good predictive value of PvCO_2_ for mortality (AUC = 0.74).^19^ However, the longitudinal trajectory of PvCO_2_ has not been analyzed. Patients with AAD, a time-sensitive disease, had dynamically fluctuating PvCO_2_ levels; therefore, studying its trajectories in a longer disease process based on longitudinal data is necessary. Compared with other AAD groups, the T3 group recorded higher rates of tracheal intubation and complications, such as heart failure, liver injury, and gastrointestinal bleeding. High CO_2_ levels lead to the internalization of Na and K-ATPase in alveolar epithelial cells, affecting the alveolar fluid clearance function.^20^ Thus, high CO_2_ levels affect lung function, leading to respiratory failure. The effects of CO_2_ on coronal circulation primarily involve coronary vasodilation due to high CO_2_, which may involve multiple mechanisms, such as local metabolic regulation, endothelial cell release of nitric oxide, and activation of ATP-sensitive potassium channels.^21^ For cardiac contractility, in patients using β-adrenergic receptor antagonists or having impaired myocardial contractility reserve, high CO_2_ levels may lead to a significant decrease in myocardial contractility. Gastrointestinal bleeding caused by a decrease in the CO_2_ level from high to low may be related to mechanisms of action, such as gastrointestinal microcirculation disorders, impaired gastrointestinal mucosal protective mechanisms, acid–base balance disorders, and local metabolic regulation. Among them, in cases such as respiratory failure, CO_2_ retention (high CO_2_ levels) can lead to increased gastric acid secretion, erosion, necrosis, ulcer formation of the gastric mucosa, and eventually gastrointestinal bleeding.^22^ A study reported that CO_2_ can increase the degradation of hypoxia-inducible factor 1 alpha in lysosomes, thereby affecting the hypoxic adaptive response.^23^ The progression pattern in the T3 group may partially reflect an initial state of respiratory failure and the subsequent progression to organ damage. This observation is consistent with our findings of higher complication rates and increased use of ventilators for respiratory support in the T3 group. Distinct pathophysiological drivers of gas trajectory in Stanford A *vs*. Stanford B.^24^,^25^

Our subgroup analysis demonstrates the fundamentally different T3 determinants between Stanford A and B AAD. For Stanford type A, the strong association between drinking and T3 suggests the dominant role of hemodynamic factors.^25^ Type AAD, involving the ascending aorta and aortic root, is highly sensitive to blood pressure surges.^26^ Chronic alcohol consumption is a well-established risk factor for hypertension and aortic wall stress.^26^,^27^,^28^ Our findings imply that T3 in patients with type A may largely reflect physiological instability driven by hemodynamic stress and lifestyle factors. Conversely, in Stanford type B, T3 was significantly associated with DBIL rather than lifestyle factors, such as drinking. Type B dissection often presents in the context of atherosclerosis and chronic degenerative changes.^29^ High DBIL levels may serve as a surrogate marker for hepatic congestion, hemolysis, or systemic metabolic stress, which could be more pronounced in descending aorta pathophysiology.^30^,^31^ This suggests that T3 in patients with type B may be primarily driven by systemic metabolic and organ dysfunction rather than acute hemodynamic fluctuations. The consistent protective role of CL across both subtypes is particularly noteworthy. Hypochloremia is a common feature of critical illness and systemic stress.^32^,^33^,^34^ The association of higher CL levels with a lower T3 risk suggests that preserved electrolyte homeostasis is a marker of physiological stability, potentially mitigating the severe gas exchange abnormalities captured by the T3 cluster.^35^ Thus, while T3 represents a distinct high-risk phenotype in AAD, its underlying triggers vary by dissection type.^36^,^37^ This heterogeneity supports the need for type-specific management strategies and underscores the importance of considering Stanford classification when interpreting gas exchange parameters in patients with AAD.

The T3 group, characterized by a continuous decline of CO_2_ from high to low levels, exhibited the poorest prognosis among all groups. This pattern may reflect a critical exhaustion of compensatory mechanisms during severe shock. Rather than indicating clinical improvement, the sustained decline suggests a collapse in circulatory reserve, resulting in persistent systemic hypoperfusion.^38^ This maladaptive state drives the progression of metabolic acidosis and tissue hypoxia. Ultimately, the cumulative burden of insufficient perfusion and metabolic derangement precipitates multiple organ dysfunction, accounting for the high mortality rate observed in this subgroup.^39^

To the best of our knowledge, this study represents the first investigation of the longitudinal PvCO_2_ trajectories of inpatients with AAD, identifying four stable AAD subclasses based on PvCO_2_ trajectories. This study boasts several strengths: to date, no study has delineated the relationship between the spontaneous trajectory of PvCO_2_ levels in patients with AAD and mortality risk, acute kidney injury, and gastrointestinal bleeding, among other adverse prognostic outcomes. However, our primary goal was to analyze 72-hour PvCO_2_ trajectories in patients with AAD and identify distinct subclasses of AAD. Additionally, an online model was created to identify the special subclass, which is challenging when relying solely on baseline characteristics, and to enhance the clinical utility and significance of our findings.

However, our findings have several limitations. First, as this study was based on a single-center database of patients who generally presented more severe conditions, its scope is inherently limited. Second, external verification was not performed. Third, the follow-up period was 6 months, with long-term follow-up planned for future studies. Fourth, the influence of other treatments, such as nutritional support, on patient PvCO_2_ trajectories remains unknown. Finally, this study did not examine responses to interventions across subclasses. Nevertheless, we aimed to deepen our understanding of the heterogeneity of AAD and successfully identify robust AAD subclasses with unique clinical characteristics and significantly different prognoses. This provides valuable insights into AAD development and paves the way for future studies focused on early subclass prediction and discovery of differential treatment responses.

In conclusion, we demonstrated the important heterogeneity of PvCO_2_ trajectories among AAD patients, highlighting the decrease PvCO_2_ level leading to the worst prognosis. A rationale for a web-based interpretable model is constructed for predicting PvCO_2_ trajectories of AAD patients with high risk postoperative adverse outcomes, implicating advantages in decision-making, stratification and treatment approaches.

## Additional files:

***Additional Table 1:*** *Subgroup analysis of prognostic factors stratified by Stanford A and Stanford B.*

***Additional Table 2:*** *Sensitivity analysis of PvCO2 trajectories stratified by admission period*

***Additional Figure 1:*** *The subgroup analysis stratified by Stanford A and Stanford B.*

Additional Figure 1 The subgroup analysis stratified by Stanford A and Stanford B.
95% CI: 95% confidence interval; Cl: chlorine; DBIL: direct bilirubin; HB: hemoglobin; OR: odds ratio.

## Data Availability

*All relevant data are within the paper and its additional files.*
